# Understanding hesitancy with revealed preferences across COVID-19 vaccine types

**DOI:** 10.1038/s41598-022-15633-5

**Published:** 2022-08-02

**Authors:** Kristóf Kutasi, Júlia Koltai, Ágnes Szabó-Morvai, Gergely Röst, Márton Karsai, Péter Biró, Balázs Lengyel

**Affiliations:** 1 Department of Economics, Rice University, Houston, TX 77005-1827 USA; 2 Centre for Social Sciences, Computational Social Science-Research Center for Educational and Network Studies, 1097 Budapest, Hungary; 3 Faculty of Social Sciences, Eötvös Loránd University, 1117 Budapest, Hungary; 4 Department of Network and Data Science, Central European University, 1100 Vienna, Austria; 5 Health and Population Lendület Research Group, Eötvös Loránd Research Network, Centre for Economic and Regional Studies, 1097 Budapest, Hungary; 6 Department of Economics, Debrecen University, 4032 Debrecen, Hungary; 7 Bolyai Institute, University of Szeged, 6722 Szeged, Hungary; 8 Alfréd Rényi Institute of Mathematics, 1053 Budapest, Hungary; 9 Mechanism Design Lendület Research Group, Eötvös Loránd Research Network, Centre for Economic and Regional Studies, 1097 Budapest, Hungary; 10 Department of Operations Research and Actuarial Sciences, Corvinus University of Budapest, 1093 Budapest, Hungary; 11 Agglomeration and Social Networks Lendület Research Group, Eötvös Loránd Research Network, Centre for Economic and Regional Studies, 1097 Budapest, Hungary; 12 Corvinus Institute for Advanced Studies, Corvinus University of Budapest, 1093 Budapest, Hungary

**Keywords:** Diseases, Infectious diseases, Psychology and behaviour, Socioeconomic scenarios

## Abstract

Many countries have secured larger quantities of COVID-19 vaccines than their population is willing to take. The abundance and the large variety of vaccines created not only an unprecedented intensity of vaccine related public discourse, but also a historical moment to understand vaccine hesitancy better. Yet, the heterogeneity of hesitancy by vaccine types has been neglected in the existing literature so far. We address this problem by analysing the acceptance and the assessment of five vaccine types. We use information collected with a nationally representative survey at the end of the third wave of the COVID-19 pandemic in Hungary. During the vaccination campaign, individuals could reject the assigned vaccine to wait for a more preferred alternative that enables us to quantify revealed preferences across vaccine types. We find that hesitancy is heterogenous by vaccine types and is driven by individuals’ trusted source of information. Believers of conspiracy theories are more likely to evaluate the mRNA vaccines (Pfizer and Moderna) unacceptable. Those who follow the advice of politicians are more likely to evaluate vector-based (AstraZeneca and Sputnik) or whole-virus vaccines (Sinopharm) acceptable. We argue that the greater selection of available vaccine types and the free choice of the individual are desirable conditions to increase the vaccination rate in societies.

## Introduction

The fast development of vaccines against the SARS-COV-2 virus will go to history books as an outstanding scientific achievement^1,2^. Unprecedented efforts and investments resulted in vaccines of various types^3^: established techniques resulted in vector-based and whole-virus vaccines while cutting-edge technologies pioneered for the mRNA vaccines. However, large populations remained hesitant against vaccination^4–9^ threatening the efficiency of vaccination plans^10–14^, especially in less developed regions^15^.

The existing literature on vaccine hesitancy considers the act of taking a vaccine as a decision under uncertainty, where the individual has to compare the potential risks and benefits of getting vaccinated^16^. As a result, the personal beliefs^17,18^ and the trusted source of information, besides demographic and socio-economic characteristics^19–21^, play a critical role in vaccine hesitancy. Previous literature shows that individuals who trust the public authorities and the scientists tend to be less hesitant^4,22,23^, while those who believe in conspiracy theories tend to be more hesitant against the vaccines^24–27^.

Despite the intensive public discourse about the vaccines’ efficiency^28^ and their potential risks^29^, the driving forces of hesitancy across vaccine types are unclear in the literature. Individuals might not be hesitant against all types of vaccines; instead, they might be open for vaccines that use certain technologies or come from certain countries^30^. To the best of our knowledge, however, the question of hesitancy by vaccine types has only been researched on theoretical grounds^31^. In this paper, we show that individual characteristics and the trusted source of information are key factors in explaining the hesitancy about particular vaccine types.

We compare the acceptance and the assessment of five vaccine types in Hungary, the country that applied the greatest diversity of vaccines during the third wave of the COVID-19 pandemic (Vaccine diversity is simply calculated by the number of available vaccine types from data at https://ourworldindata.org/covid-vaccinations). Besides the centralized European Union (EU) purchase, which included the Pfizer-Biontech, Moderna and AstraZeneca vaccines, the Hungarian government imported Sputnik from Russia and Sinopharm from China. Although Janssen was also available in Hungary from May 2021, only a small share of population received it. We excluded this vaccine from the analysis (Official names of the vaccines are listed here. **Pfizer-Biontech**: Comirnaty/BNT162b2, COVID-19 mRNA vaccine. **Moderna**: Spikevax/mRNA-1273. **AstraZeneca**: Vaxzevria, Covishield/AZD1222, ChAdOx1 nCoV-19, ChAdOx1-S, AZD2816. **Sputnik**: Gam-COVID-Vac. Sinopharm: **Sinopharm**: BIBP COVID-19 vaccine/Zhong’aikewei, Hayat-Vax. **Janssen**: Ad26.COV2.S JNJ-78436735 Ad26COVS1 VAC31518). The availability of Sputnik and Sinopharm in the early vaccination period put Hungary among countries with the highest vaccination rate globally. The vaccination rate was still outstanding among Central and Eastern European countries at the beginning of the fourth wave (According to https://ourworldindata.org/covid-vaccinations, vaccination per capita was much higher in May 2021, when our data was collected, in Hungary than in other Central and Eastern European countries where trust in public authorities is at a similar level). The government strongly communicated the safety and efficiency of Sputnik and Sinopharm^27,32^; while mRNA vaccines were highly rated in the society^33^. As hesitancy was high in the beginning of the vaccination campaign^22^ ($53\%$ in January 2021 and $20\%$ in May 2021^33^), the government introduced a more flexible vaccine assignment policy to increase people’s willingness to vaccinate. As a result, individuals could reject the assigned vaccine and wait for a more preferred alternative.

A vaccine seeking individual (patient) had to go through the following procedure in Hungary over the third wave of the COVID-19 pandemic in the Spring of 2021. First, the patient had to register online at a government portal. The general physician (GP) received the list of patients in its region and contacted them in an order of priority. The priority was determined based on chronic illness, age and occupation. Upon contacting a patient, the GP offered a type of vaccine to the patient. The offer depended on patient characteristics (as explained above) and the availability of vaccines. The patient could either accept or reject the offered vaccine. In case of a rejection, the GP had to reach out to the patient later, when a different type of vaccine became available. This procedure characterized early vaccination until the vast majority of priority groups were vaccinated. Starting from April, when vaccines became increasingly available, patients could register and select available vaccines through a web-application (first AstraZeneca, then Sinopharm and Sputnik, and later other vaccines as well). Besides giving floor for various individual preferences, the Hungarian mechanism might have provided acceptable alternatives for those who got scared from the offered vaccine. For example, the Sinopharm vaccine was offered initially in Hungary to the older generation, despite it was recommended to a younger population. Similarly, AstraZeneca was offered for the middle-aged generation, but many became afraid when the connection between the vaccine and thrombosis cases were discussed.

This historically unique situation enables us to contribute to the ongoing discussion in the following ways. First, we can compare hesitancy across vaccines by technologies (mRNA, vector-based, and whole virus). Second, we are able to identify the individual characteristics that correlate with hesitancy of specific vaccines, such as the role of trusted source of information. Third, we can analyze the role of free vaccine choice for vaccine hesitancy due to the special allocation mechanism.

## Results

The empirical analysis is based on a nationally representative survey (N = 1000) that we conducted in Hungary at the end of the third wave of the pandemic when vaccines were already available for everyone (Number of cases can vary among analyses. For a precise consideration of sample sizes, please see “Methods”). Data collection is described in “Methods”.

### Vaccine assignment and revealed preferences

In our survey, we focused on demographic, socio-economic, and health related questions that could help to understand COVID-19 vaccine hesitancy better. Specifically, the participants had to evaluate each vaccine, identify their trusted sources regarding the vaccination and state whether their assessment has changed over the third wave period. Then, they were asked a list of questions about their COVID-19 infection and their vaccination history including the date of potential positive tests, date and type of the received and the rejected vaccines. This latter information provides new insights into the nature of vaccine hesitancy through the analysis of revealed vaccine preferences. From now on, $evaluate$ refers to whether an individual considers the vaccine types acceptable, $reject$ refers to the act of not willing to take the assigned vaccine and $accept$ refers to the act of taking the assigned vaccine.

**Table 1:** Percentage of individuals in the sample by major socio-demographic characteristics and COVID vaccination history. Percentages in the last three columns add up to 100$\%$ by character groupings (except the rows on specific vaccine types where the last column is not relevant). In the last section of rows, we classified the individuals by the accepted vaccine type.

	Evaluated all vaccines unacceptable (%)	Evaluated one vaccine acceptable but another unacceptable (%)	Vaccinated without rejection (%)	Vaccinated after rejection (%)	Non-vaccinated (%)
Men	1.1	7.9	30.7	4.6	11.7
Women	1.2	10.3	32.2	6.2	14.5
University	0.2	3.4	14.4	2.9	4.3
High-school	1.7	11.7	32.7	6.2	14.7
Elementary	0.4	3.1	15.9	1.7	7.2
Not chronic Illness	1.6	11.8	33.0	6.4	19.9
Chronic Illness	0.7	6.4	29.9	4.4	6.3
Age 20–39	0.9	7.8	16.2	4.3	12.6
Age 40–59	0.8	5.9	20.6	3.4	9.2
Age 60–79	0.5	4.0	23.0	2.7	3.3
Age 80+	0.0	0.2	2.3	0.0	0.3
Pfizer	−	5.0	20.3	5.8	–
Moderna	−	0.8	3.5	1.0	–
AstraZeneca	−	1.1	12.1	1.1	–
Sputnik	−	1.4	12.3	1.3	–
Sinopharm	−	1.5	14.7	1.6	–

Table 1 describes the vaccination related variables by socio-demographic characteristics of the respondents. The evaluation and the rejection of vaccines—which information makes our dataset unique—are presented by sex, education level, chronic illness, and age categories. Here we can distinguish those who rate all vaccine types unacceptable ($2.3\%$) from those who evaluate at least one vaccine unacceptable but evaluate another acceptable ($18.2\%$). Furthermore, we can compare those who accepted the first vaccine offered ($62.9\%$) with those who rejected the first offer ($10.8\%$). Apparently, the women, the younger cohorts, the lower educated and those with no chronic disease are more likely to reject a vaccine offer in general. We also find that $5\%$ of those respondents who received Pfizer rejected at least one other vaccine. These shares are lower for other vaccine types. Furthermore, we find that $20.3\%$ of all respondents have accepted Pfizer without rejecting any vaccines while $5.8\%$ waited for Pfizer after rejecting another vaccine.

**Figure 1:**
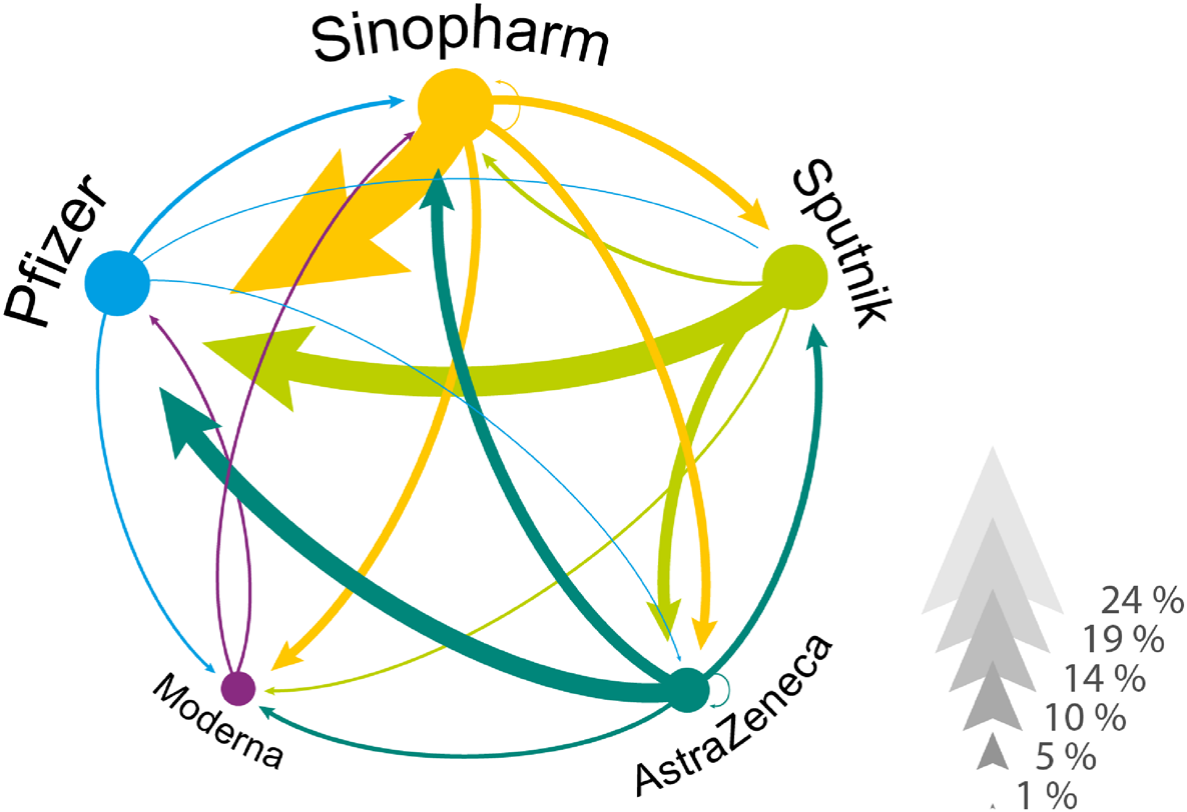
Revealed vaccine preferences by rejection and re-selection. The network of revealed vaccine preferences in Hungary. Vaccines are linked if someone in our data has rejected one vaccine and later accepted another. The direction of the link goes from rejected to accepted and the width of the arrow corresponds to the percentage of individuals who acted accordingly. We conditioned on those individuals who rejected at least one vaccine.

Figure 1 illustrates the network of revealed preferences across vaccine types from our data. Focusing on those who rejected a vaccine (in total, 18% of our vaccinated respondents rejected an assigned vaccine and later took another type), we observe a high ratio of individuals, who received Pfizer but rejected Sinopharm ($23\%$), Sputnik ($15\%$) or AstraZeneca ($9.7\%$). We also see that a few individuals rejected an mRNA vaccine to accept a less popular vaccine later. For example, $2.2\%$ of the hesitant population rejected Pfizer to receive Sinopharm.

**Figure 2:**
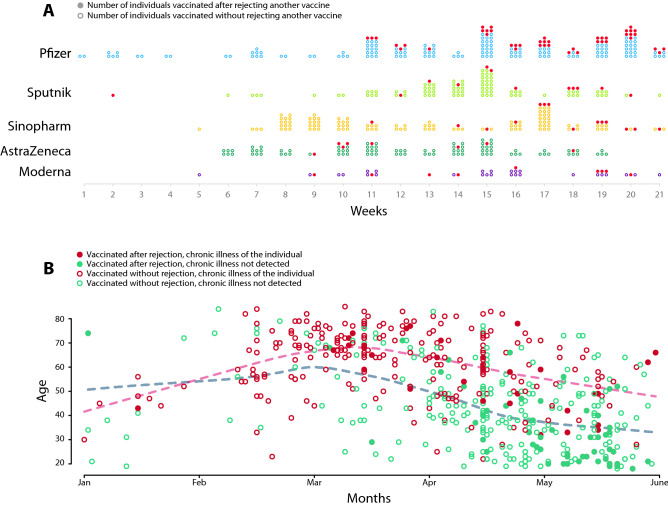
Dynamics of vaccination by vaccine types and re-selection. (**A**) Dynamics of vaccination by vaccine types. Dots represent the taken vaccine by weeks in 2021. Dark dots represent the vaccines that were accepted after rejecting another one. (**B**) The dynamics of vaccination by age and chronic illness. We used red circles for the chronically ill patients and green circles for the healthy individuals to show the day of receiving the first vaccine. Filled markers denote the individuals who rejected at least one vaccine earlier. Dashed lines represent the local averages of vaccinated patients’ age.

Figure 2A demonstrates the dynamics of revealed preferences by vaccine types. The Pfizer was the first vaccine that patients could receive in Hungary starting at the first week of 2021. The Sputnik, the Sinopharm and the AstraZeneca only became available in larger quantities after week 5 of 2021. Most of the Moderna vaccines were accepted after week 9 of 2021. Figure 2B shows the time of the first vaccine and the age of the patients. We grouped the individuals based on chronic illness status and whether they rejected a vaccine in the past. We find that those chronically ill, elder patients who rejected vaccines received a more preferred vaccine in March. The younger healthy individuals who rejected a vaccine received a more preferred vaccine only after mid April. Supporting Information 1 contains further description of the dynamics of vaccination and rejection by age, chronic illness and vaccine types.

### Trusted information sources characterize groups of vaccine preferences

To observe hesitancy by vaccine types, we asked respondents to evaluate vaccine types on a scale from 1 “unacceptable” to 5 “best”. For the reasons of simplicity, here we code all evaluations different from 1 as “acceptable” and analyse rating details in section “Individual selection improves vaccine assessment”. Supporting Information 2 illustrates that Pfizer and Moderna were more likely to get accepted than Sputnik, which is significantly more likely to get accepted than AstraZeneca and Sinopharm. Chi square tests imply that the distributions of vaccine acceptance are not independent from each other. Instead, acceptance is correlated across vaccine types, and especially between Pfizer and Moderna and between Sputnik and Sinopharm.

We use multivariate regression analysis to investigate the heterogeneity of vaccine evaluation by types and its association with the source of trusted information. This latter determinant was used in the previous studies to explain vaccine hesitancy^25^, while other research focused on the communication channels that individuals collected vaccine-related information from^18,34^. Accordingly, respondents were asked in our survey to confirm if they follow the vaccine-related advice of doctors, scientists, anti-vaccine propagators, politicians, family, friends, journalists and celebrities. These major actors communicated differently about the vaccines (Doctors and scientists argued for vaccination, see for example the recommendations of Hungarian Academy of Sciences: https://mta.hu/english/recommendation-of-the-hungarian-academy-of-science-on-the-management-of-the-covid-19-epidemic-in-the-short-and-long-term-110630. Anti-vaccine propagators emphasized the non-effectiveness and the side effects of the COVID-19 vaccines. A summary on anti-vax movement in Hungary can be found at https://www.iribeaconproject.org/our-work-analysis-and-insights/2021-09-16/hungarys-anti-vax-movement-alive-and-kicking. Some medical doctors warned against vaccines that are not approved by the EU, while others urged to vaccinate with any vaccine: https://www.reuters.com/article/us-health-coronavirus-hungary-vaccines-idUSKBN2CE2C3. The government communication campaigned for the non-EU-approved vaccines to improve their rate of acceptance by the population: https://www.euronews.com/2021/02/28/hungary-s-pm-viktor-orban-vaccinated-against-covid-with-chinese-sinopharm-vaccine. Yet, a heated debate around vaccine types has fragmented the society: https://hungarytoday.hu/hungarian-vaccination-politics-vaccine-preference-coronavirus-survey-willingness/). The respondents also had to name their most frequently used information channels on pandemic measures such as web news, social media, press, radio, TV, family and friends. These explanatory variables are complemented by demographic and socio-economic characteristics of respondents^25^. We apply a two-step procedure in the regression analysis. First, we use a machine learning technique to select the most important variables. Second, we run the regressions using the selected variables.

In the first step, we apply a variable selection method. This is important because the source of trusted information and the channel of communication can influence hesitancy simultaneously. The LASSO model, which is a short notation for the least absolute shrinkage and selection operator, enables us to select the most important variables that have high-enough predictive power. We describe the process in the “Methods” section in greater details. The estimation results are reported in Supporting Information 3, Table S2. We find that communication channels are not important determinants of hesitancy as all but one (web news) are dropped from the model. However, we see that both the demographic characteristics and the sources of trusted information on COVID-19 vaccination are important predictors of the outcome variables. We rely on this result when we select the explanatory variables in the linear probability models.

**Figure 3:**
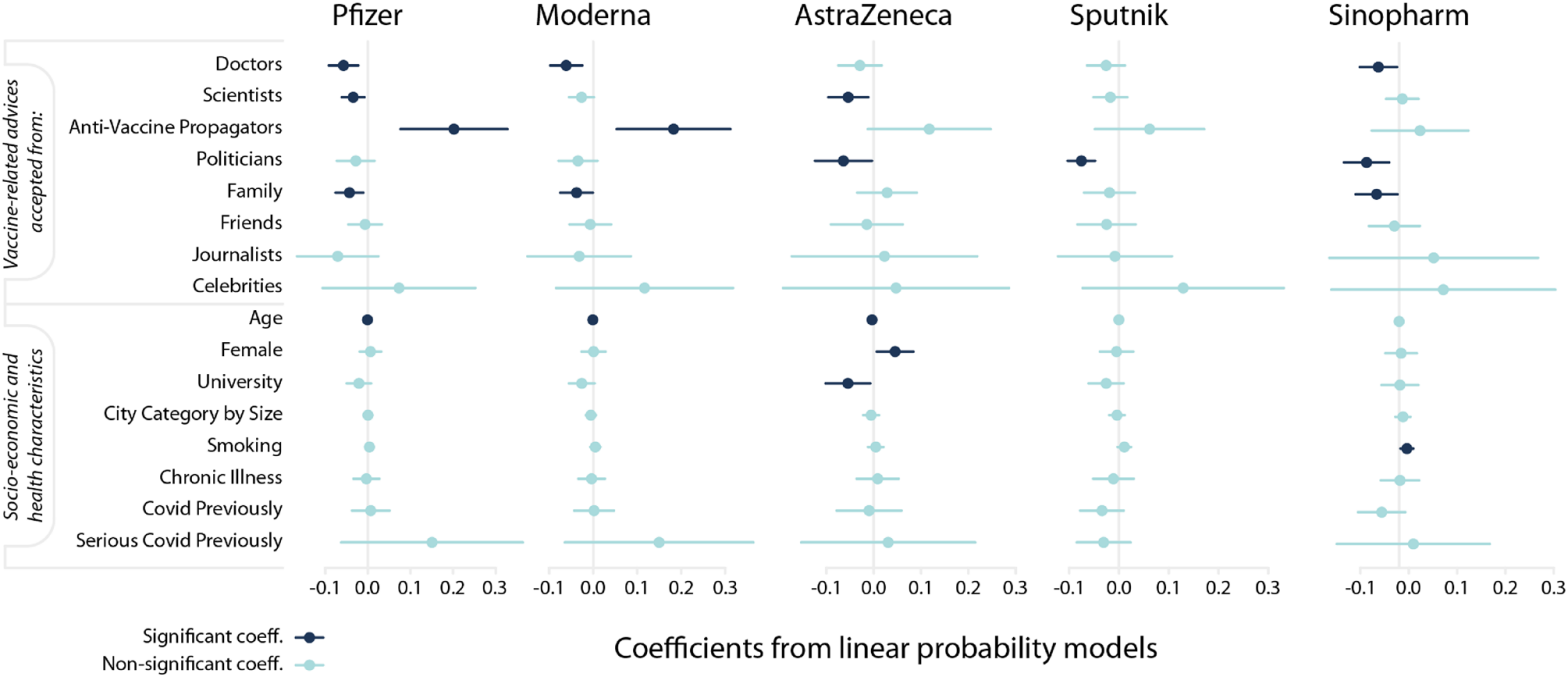
Source of advice taken characterize hesitancy against vaccines differently. Estimation results from linear probability models with robust standard errors. The dependent variable equals 1 if the respondent evaluates a vaccine type unacceptable and 0 otherwise. Markers denote point estimates and 95% confidence intervals are denoted by the bars. Non-significant coefficients are in light blue ($p\ge 0.05$); significant coefficients are in dark-blue ($p<0.05$).

Next, we estimate linear probability models (LPM) to analyze how different factors shape the evaluation of each vaccine individually. Independent variables include “Advice” dummy variables that take value 1 if the individual follows the advice of the denoted source. “Age” is a numeric variable; it is measured in years and is expected to correlate negatively with hesitancy. “Female” takes value 1 if the respondent is woman and zero otherwise. “University” equals 1 if the respondent has a university degree or equivalent and is expected to correlate negatively with hesitancy. We include health-related information as well. “Smoking” and “Chronic Illness” are binary variables as well as “COVID Previously” and “Serious COVID previously”. We present the summary statistics in Supporting Information 4, which also contains a correlation matrix and VIF statistics. The latter tests indicate no issues of multicollinearity. The estimation specification can be found in “Methods”.

Figure 3 shows the point estimates and the $95\%$ confidence intervals calculated from the heteroscedasticity-robust standard errors (for detailed results see Table S3 in Supporting Information 5). The results suggest that the driving factors of rejecting a decision differ among vaccine types. Those who trust the advice of anti-vaccine propagators are 18.2 and 20.2 percent more likely to evaluate mRNA vaccines (Pfizer and Moderna) unacceptable. Trusting the doctors’ advice, however, decreases the hesitancy probability by 5.7, 6.1, and 5.4 percent for Pfizer, Moderna, and Sinopharm, respectively. Trusting scientists reduce the likelihood of evaluating a vaccine unacceptable by 3.5, 2.6 and 5.5 per cent for Pfizer, Moderna, and AstraZeneca, respectively. Those who take advice from politicians are 6.4, 7.6 and 8.4 percent more likely to evaluate AstraZeneca, Sputnik and Sinopharm acceptable, respectively. Those who trust their family’s advice are more likely to evaluate Pfizer, Moderna and Sinopharm acceptable by 4.3, 3.8 and 5.8 percent, respectively. The coefficients for the advice of celebrities are either not or only slightly significant. We find that the point estimates are large and positive for all types of vaccines.

Socio-demographic characteristics of respondents correlate with vaccine hesitancy in the following way. Age has negative correlation implying that more vulnerable, older individuals are less likely to evaluate vaccines unacceptable. Although the coefficient of age is small in Fig. 3, the regression on standardized variables reported in Table S3 (in Supporting Information 4) reveals that age is an important factor for evaluating Pfizer, Moderna and AstraZeneca acceptable. Women are more likely to evaluate AstraZeneca unacceptable, probably due to reported cases of females’ blood clot (https://www.reuters.com/business/healthcare-pharmaceuticals/uk-regulator-says-some-evidence-astrazeneca-clots-occur-more-women-than-men-2021-05-06/). Note that a meta-analysis^9^ found an overall significant gender difference with males being on average 41% more likely to report that they intended to receive a vaccine than females. Our results can indicate that this extra hesitancy for females might come from the different risk-tolerance regarding serious side-effects. University degree, however, decreases hesitancy towards AstraZeneca.

We have carried out the following robustness checks in Supporting Information 5. First, we show that our regression results hold if we use a logistic regression specification instead of ordinary least squares (both of these specifications have been used previously to examine vaccine acceptance^35^). Second, our results remain similar after including subjective wealth as a control variable. A potential limitation of our study could be from “spreading of the alternatives”^36^, which means that after a difficult decision making, individuals tend to value the accepted alternative better and the rejected alternative worse. However, our results do not change if we apply the models on a subsample of those who stated that they had stable vaccine assessment over the time.

### Individual selection improves vaccine assessment

**Figure 4:**
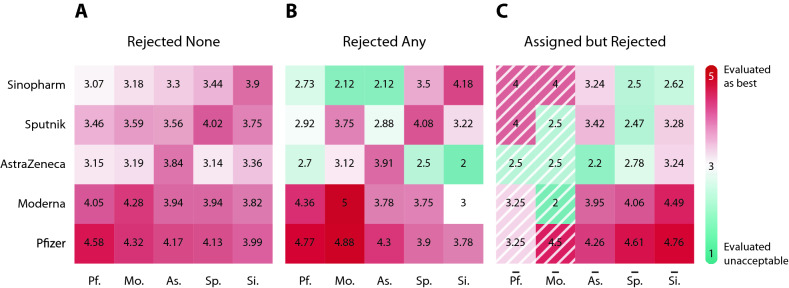
Average vaccine rating by assigned, re-selected and rejected vaccines show polarization of vaccine hesitancy. Individuals are grouped by columns of received vaccine in (**A**) and (**B**) and by columns of assigned vaccine in (**C**) (marked by overline). Rows correspond to average rating of a certain vaccine. (**A**) Ratings of those patients who accepted their first vaccine offer suggest that Pfizer and Moderna were the best assessed vaccines. (**B**) Ratings of those patients who rejected at least one assigned vaccine reveal that re-selection of vaccines is paired with better assessment of the received vaccine. (**C**) Ratings of those patients who rejected at least one assigned vaccine illustrate polarization of hesitancy by vaccine types. The average rating in the first two shaded columns are calculated from few observations only.

To better understand how free choice across vaccines is related to their acceptance, we analyze individuals’ vaccine evaluation values on a scale from $1$ to $5$ sorted by the received and the rejected vaccines. Figure 4 summarizes the vaccine evaluations in the form of a heat map. A heat map cell corresponds to the average rating of a type of vaccine. The row denotes the average evaluation for that vaccine among the vaccine group of patients denoted by the column. Supporting Information 6 contains summary statistics of vaccine assessments depicted in Fig. 4.

In Fig. 4A, we consider individuals who accepted their first vaccine offer. Notice that Pfizer has the highest average ratings across all columns. This implies that free vaccine choice does not necessarily mean that everyone gets the most preferred vaccine. Instead, individuals make an uncertain decision considering the acceptable alternatives and the unknown waiting times between the sequential vaccine offers. In the Supporting Information 7, Table S13 presents the distribution of the most preferred vaccine when the accepted vaccine is not the most highly-rated vaccine. We infer that patients were willing to accept a vaccine other than the preferred Pfizer or Moderna to avoid the long anticipated waiting times. In Table S14, we show that patients had to wait almost $2.76$ weeks on average to get their most preferred vaccine if they rejected a less preferred vaccine. In Table S15, we also present evidence that the anticipated waiting times closely follow the actual waiting times on average. That is, if a patient accepts the first vaccine offer then the following conditions must hold; the patient prefers the accepted vaccine to the other vaccines **or** she has a belief that a more preferred vaccine, which is likely to be Pfizer and Moderna, will not be offered anytime soon. We provide an extended analysis in the Supporting Information 7.

Figure 4B shows the ratings of those patients who rejected at least one vaccine. We see that the diagonal values in Fig. 4B are slightly higher than the ones in Fig. 4A. This implies that the individuals who rejected a vaccine before value their received vaccine slightly higher than those who accepted their first vaccine. Figure 4B also shows a high level of dislike towards AstraZeneca, Sinopharm and somewhat Sputnik when it comes to rating vaccines other than the accepted vaccine.

Comparing the values on Fig. 4A,B, we can see fundamental differences between the rejected none and rejected any groups. Based on the values on the diagonal, those individuals who rejected at least one vaccine rated the received vaccine higher than those who accepted the first vaccine offer. Looking at the off-diagonal, we see that the rejected any group tends to rate the non-accepted vaccines to be worse. For instance, the mean ratings for AstraZeneca at the off-diagonal are strictly smaller among those individuals who rejected a vaccine at least once than among those who accepted their first offer. We can also see the individuals who received Sinopharm after rejection tend to rate other vaccines worse than those who accepted Sinopharm in their first offer.

Figure 4C highlights the benefits of free choice. We group individuals based on the first vaccine offer rejection denoted by the columns. We implicitly assume here that the vaccine registration is not affected by the government’s decision to allow free choice. Let us point out that the number of observations is small for the Pfizer and the Moderna groups with only $6$ and $2$ observations, respectively. In other words, patients rarely reject Pfizer or Moderna when these vaccines are offered for the first time. Focusing on the AstraZeneca, Sputnik and Sinopharm columns, we see that the values for the assigned vaccines on the diagonal are strictly smaller than any of alternatives. We can also see a strong preference towards Pfizer and Moderna from the AstraZeneca, the Sputnik and the Sinopharm columns. Figure 4C suggests that free choice significantly increased the efficiency of vaccine allocation. This follows from enabling the re-selection of acceptable vaccines.

We report how assessment of vaccines have changed over the third wave in Supporting Information 7. The ratio of patients who changed their assessment over time for a specific vaccine stays below 5 percent of the total sample. We can only see a large negative change in the case of the AstraZeneca reaching 10 percent of the sample. This is most likely a result of the news about the possible side effects. Finally, Supporting Information 8 contains a preference analysis of those individuals who were not vaccinated but evaluated minimum 2 but maximum 3 of the investigated vaccines acceptable. This enables us to look at co-acceptance among vaccines. In Fig. S7 we find that the most common co-acceptance link is between Pfizer and Moderna. The Sputnik-Sinopharm co-acceptance link is the most frequent one that does not include Pfizer. This suggests that a wide and diverse vaccine portfolio is preferred in a society where vaccine preferences diverge.

## Discussion

Most European countries did not allow individuals to choose between the available vaccines in the first half of 2021. An exception is the case of Spanish essential workers who were allowed to choose AstraZeneca or Pfizer for the second dose^37^. However, the potential benefits of free vaccine choice have been addressed in the scientific discussion^38,39^. The main reason for these benefits is that free choice can fulfill heterogeneous individual preferences that in theory, may differ by the technology and the country of origin^30^. Experiments carried out in Germany have indeed found evidence that free choice across Pfizer and AstraZeneca, the two most different and also the most preferred vaccines available in the country in April 2021, has mitigated vaccine hesitancy^40^. Another study has shown that the vast majority of Italian college students preferred the mRNA technology to vector vaccines^41^. A recent survey conducted in eight European countries shows the differences in vaccine hesitancy and the divergent preferences over the available vaccines^42^.

We add to this line of research by analyzing a representative survey in Hungary. This survey not only asks the preferences of the patients, but also collects information about their actual decisions over accepting or rejecting specific vaccine offers. Hence, the survey participants provide their revealed preferences over the available vaccines.

Our results imply heterogeneous vaccine preferences. Most individuals prefer mRNA vaccines such as the Pfizer and the Moderna. However, a non-negligible portion ($5.5\%$) of respondents who rejected an mRNA vaccine preferred a vector-based or a whole virus vaccine. We find evidence for divergent attitudes towards vaccine types depending on the trusted source of advice. Those who trust politicians are more likely to accept less popular vaccines such as the vector-based (AstraZeneca and Sputnik) and the whole-virus vaccines (Sinopharm). Those who are open to conspiracy theories are more likely to reject mRNA vaccines (Pfizer and Moderna) that are the most preferred vaccines in the country. We show that the rejection of non-desired and the re-selection of preferred vaccines generally improve the assessment of the received vaccine.

These findings suggest that the free choice policy in vaccine allocation should be combined with the right communication strategy by the government to achieve higher vaccination rate in societies. In early stages of vaccination campaigns, such as the one during the third wave of the COVID-19 pandemic, vaccines are hard to evaluate. Competing expert rankings of vaccine technologies, country of origin, effectiveness, side effects, approval status by national and international authorities can yield very diverse recommendations^43^. Consequently, individual hesitancy can vary by vaccine types. The diverse preferences which are present in societies^15,42^ can change over time^30^. At times when the scientific evidence about side-effects and effectiveness of vaccines are not available^44^, the governments and the scientific community must earn the trust of the society. Helping individuals to find their most preferred vaccines and to allow the free choice across vaccine types are therefore desirable for future vaccination campaigns.

Since our results are based on a survey, we might underestimate the rate of the hesitant population due to the social desirability bias^45^. Those who are more concerned about the COVID-19 situation and more willing to answer the survey are also more likely to accept a vaccine. Furthermore, due to social pressure some may expressed no hesitancy in the survey, whilst in reality they have never taken any vaccine^46^.

## Methods

### Data collection

The data used in this paper was collected with a CATI telephone survey on a nationally representative sample of the Hungarian adult (18 years or older) population between May 25–31, 2021, which corresponds to the end of the third wave of the pandemic. The questionnaire was designed by the authors with diverse expertise, including epidemiological mathematicians, network scientists, economists, economic geographers and sociologists—all working with COVID-19 related research. The sample size is 1000, which is equal to the conventional sample size for nationally representative samples in the country. The data is representative for the adult Hungarian population by gender, age, education and domicile. Data was collected both over mobile and landline phones. The public opinion research company, who was responsible for the data collection, controlled data quality by comparing the voice responses with the recorded answers on randomly selected calls. This campaign was a part of a larger data collection effort, which started in March 2020 and it is still ongoing. For more details about the data collection effort, see^47^. The sample was selected by a multi-step, proportionally stratified, probabilistic sampling procedure. Minor deviations of the data from population ratios were corrected by iterative proportional weighting after the data collection. After data collection, only the anonymised and hashed data was shared with people involved in the project after signing non-disclosure agreements. The data collection was fully complying with the actual European and Hungarian privacy data regulations and was approved by the Hungarian National Authority for Data Protection and Freedom of Information^48^, and also by the Research Ethic Committee of the Medical Research Council of Hungary (resolution number IV/3073-1/2021/EKU). Informed consent was obtained from all survey participants.

### Statistical analysis

In the LASSO model, the dependent variable takes values between 1 and 5, where 1 is “unacceptable” and 5 is “the best”. In models 1–5 presented in Supporting Information 3, we estimate evaluation values by vaccine types. Then, in models 6 to 9, the ratings’ variance, mean, minimum, and maximum serve as dependent variables. In model 10, the dependent variable is binary taking the value of 1 if ever rejected a vaccine offer and 0 otherwise. The dependent variable is 1 in the model, if any vaccine is unacceptable. The lasso coefficients ($\widehat{\beta }_{\lambda }^{L}$) minimize the following expression: $\sum _{i=1}^{n}(y_{i}-\beta _{0}-\sum _{j=1}^{p}\beta _{j}x_{ij})^{2}+\lambda \sum _{j=1}^{p}\left| \beta _j \right|$ .

Linear probability models are used to predict rejection of vaccines. These regressions are specified by the formula $P(Y=1|X)=\alpha + \beta X + \varepsilon$, where *Y* equals 1 if the vaccine is rated unacceptable, $\alpha$ is the intercept, $\beta$ is point estimates, *X* is a vector of independent variables, and $\varepsilon$ is heteroscedasticity-robust standard error.

To generate the heat maps, we used the same vaccine ratings on a scale from $1$ to $5$, as we mentioned above. We clustered the survey responses based on the acceptance decision of the first vaccine offer. In case of rejection, we grouped individuals based on the accepted vaccine and the first rejected vaccine. Then, we used standard summary statistics tools to find the number of observation, mean and the standard deviation for each of the groups. We report the mean ratings in Fig. 4 and the rest of the summary statistics in Supporting Information 6.

The link weights of the directed network presented in Fig. 1 is generated following the aggregation procedure $W_{ab}=\sum _{a}L_{ab}$, where $L_{ab}$ equals 1 if the individual has rejected vaccine *a* but later received vaccine *b*. The undirected co-occurrence network presented in Fig. S7 follows the rule of $W_{mn}=\sum _{m}L_{mn,m \ne n}$ where $L_{mn}$ equals 1 if the respondent accepts both vaccines *m* and *n*.

All methods were performed in accordance with the guidelines and regulations of the Hungarian National Authority for Data Protection and Freedom of Information^48^ and were approved by the Research Ethic Committee of the Medical Research Council of Hungary (resolution number IV/3073-1/2021/EKU).

## Supplementary Information

Supplementary Information.

## Data Availability

For privacy protection we share only the relevant part of the data, which does not allow the reidentification of participants. We exclude sensitive information such as domicile and labels for categorical responses. The data is available at https://doi.org/10.5281/zenodo.5575586.

## Code availability

All codes to analyze data have been written in R (version 4.0.5) and are available at https://doi.org/10.5281/zenodo.5575586.
