# Supplementary material for: Understanding hesitancy with revealed preferences across COVID-19 vaccine types
Source: Sci Rep. 2022 Aug 2;12:13293. doi: 10.1038/s41598-022-15633-5 (PMC9345393; doi:10.1038/s41598-022-15633-5)
Supplement: Supplementary file 1 — Supplementary Information. [file 41598_2022_15633_MOESM1_ESM.pdf]

Supplementary Information for Understanding hesitancy with revealed preferences across COVID-19 vaccine types

Kristóf Kutasi[1], Júlia Koltai[2,3,4], Ágnes Szabó-Morvai[5,6], Gergely Röst[7], Márton Karsai[4,8], Péter Biró[9,10,+], and Balázs Lengyel[11,12,+,*]

[1]Rice University, Department of Economics, Houston TX, 77005-1827, USA
[2]Centre for Social Sciences, Computational Social Science - Research Center for Educational and Network Studies, Budapest, 1097, Hungary
[3]Eötvös Loránd University, Faculty of Social Sciences, Budapest, 1117, Hungary
[4]Central European University, Department of Network and Data Science, Vienna, 1100, Austria
[5]Eötvös Loránd Research Network, Centre for Economic and Regional Studies, Health and Population Lendület Research Group, Budapest, 1097, Hungary
[6]Debrecen University, Department of Economics, Debrecen, 4032, Hungary
[7]University of Szeged, Bolyai Institute, Szeged, 6722, Hungary
[8]Alfréd Rényi Institute of Mathematics, Budapest, 1053, Hungary
[9]Eötvös Loránd Research Network, Centre for Economic and Regional Studies, Mechanism Design Lendület Research Group, Budapest, 1097, Hungary
[10]Corvinus University of Budapest, Department of Operations Research and Actuarial Sciences, Budapest, 1093, Hungary
[11]Eötvös Loránd Research Network, Centre for Economic and Regional Studies, Agglomeration and Social Networks Lendület Research Group, Budapest, 1097, Hungary
[12]Corvinus University of Budapest, Corvinus Institute for Advanced Studies, Budapest, 1093, Hungary
[*]Corresponding author: lengyel.balazs@krtk.hu
[+]these authors jointly supervised this work

Supplementary Information

Supporting Information 1: Detailed information on vaccination dynamics

Figure S1 illustrates the time horizon for the vaccination. We can see that Pfizer was the only available vaccine initially, which was most likely offered to medical workers. In the beginning of March, Sinopharm and AstraZeneca became available for mostly chronic ill patients. We see a strict cutoff at the age of 60, where AstraZeneca was offered to patients below 60 and the Chinese Sinopharm to those who are above 60. Chronic ill patients who rejected a vaccine get vaccinated in the end of March. In April, we see that vaccines become widely available for patients under the age of 50. In May, those patients who have rejected a vaccine before and are not chronic ill get finally vaccinated, mainly with Pfizer. Figure S1 suggests that chronic ill and older patients who have not rejected any vaccines in the past tend to get vaccinated sooner.

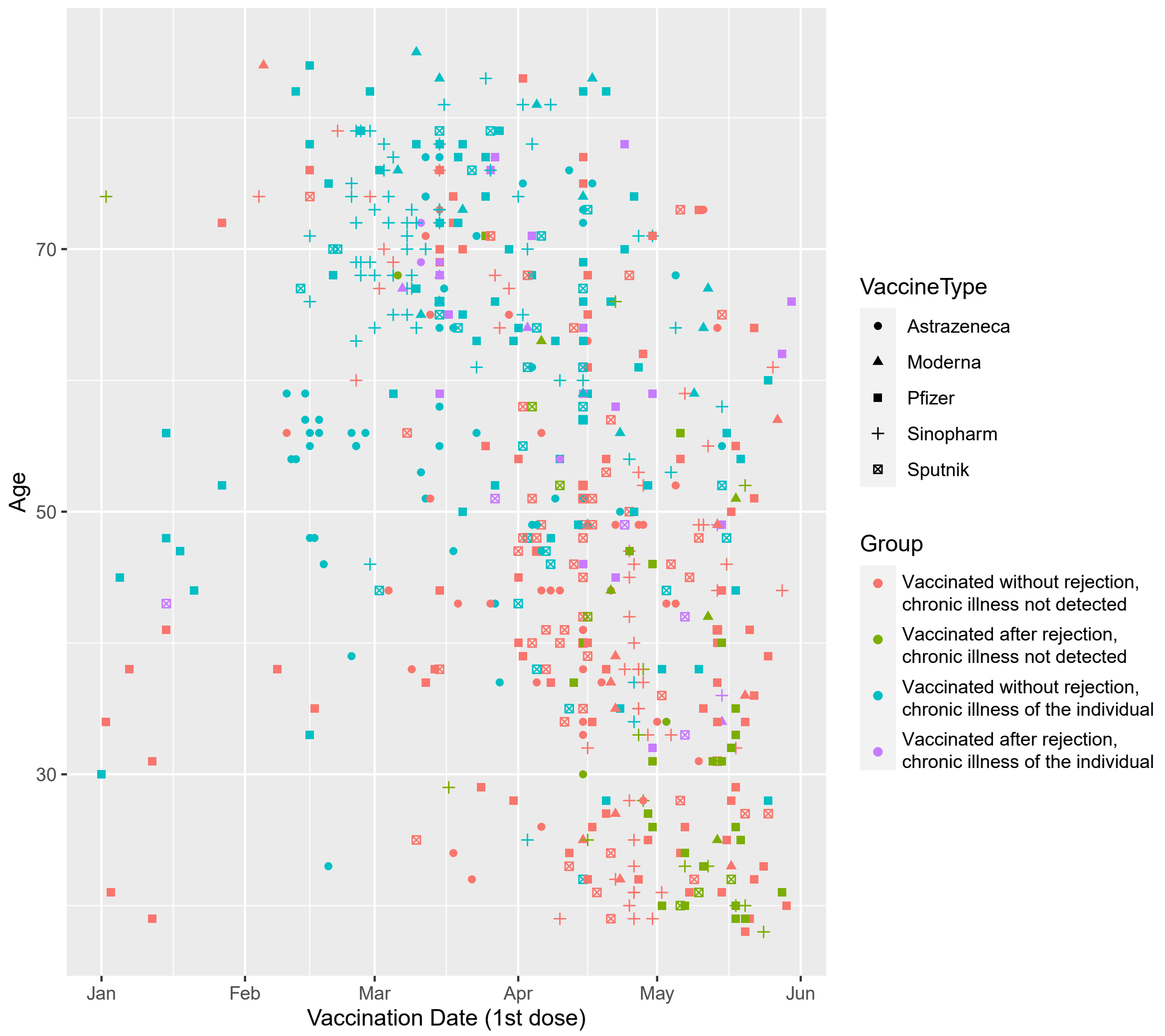

Figure S1: Vaccination date, age, vaccination type, chronic illness and rejection status distribution

Figure S2 shows the distribution of different vaccine types conditionally on chronic illness and rejection status. Out of the 996 observations, most patients received Pfizer ($n = 260$), then Sinopharm ($n = 162$), Sputnik ($n = 136$), AstraZeneca ($n = 122$), Moderna ($n = 45$) and $n = 261$ people decided not to get vaccinated. We see that only a minority of patients rejected any vaccines. We would like to highlight that Pfizer and Moderna had the highest and AstraZeneca had the lowest ratio of patients who rejected a vaccine before vaccination. We would also like to point out that Sputnik and Pfizer have the two highest not chronic ill to chronic ill ratio of patients. More importantly, Figure S2 suggests that chronic ill patients are less likely to reject a vaccine than patients without any chronic illness.

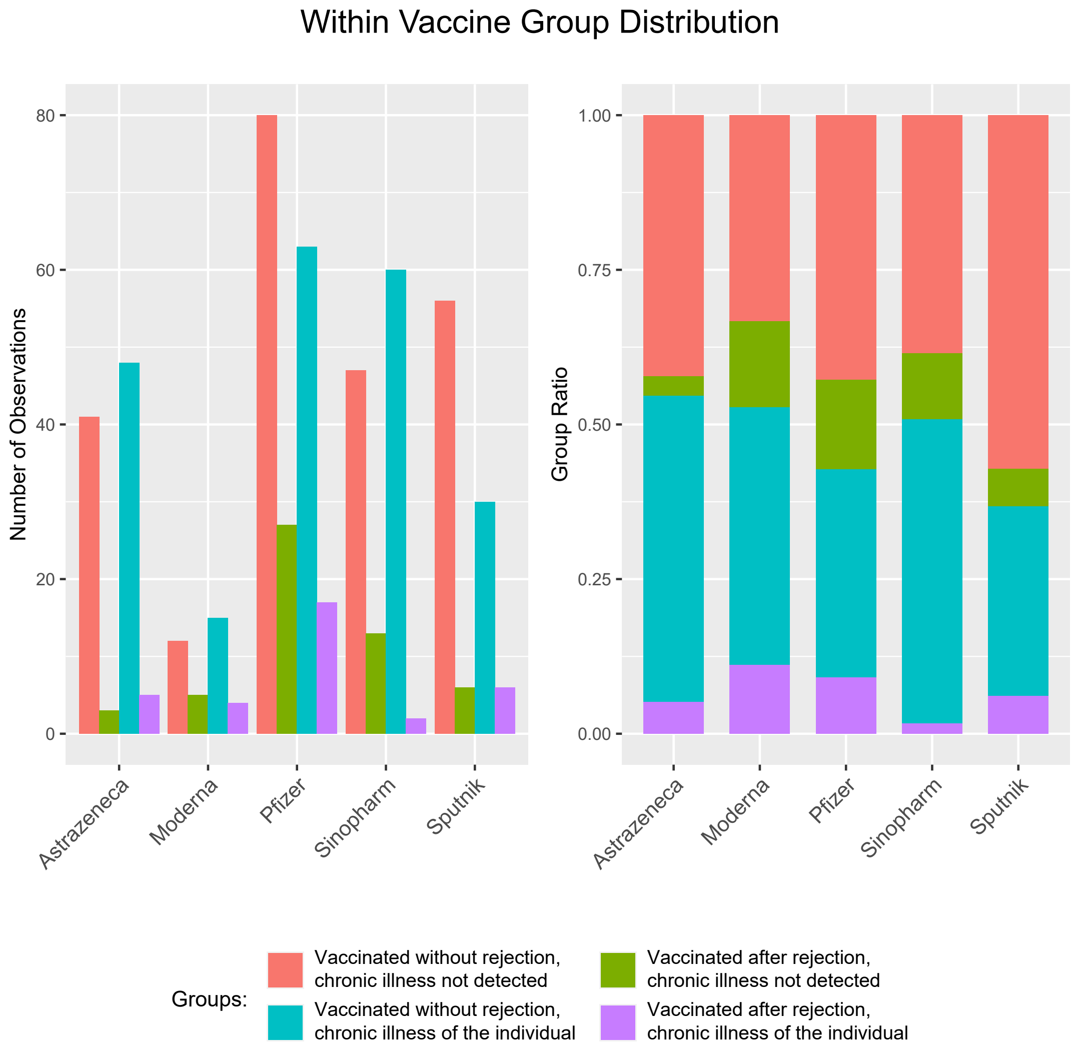

Figure S2: Vaccine type distribution conditionally on chronic illness and rejection status.

Figure S3 shows the mean and standard deviation of the age distribution conditionally on vaccine type, chronic illness and rejection status. We see that chronic patients tend to be older, as expected. Among patients without chronic illness, Pfizer has the lowest average age, which we believe is a consequence of 2 reasons. First, younger medical workers and school teachers could receive Pfizer sooner, as they were prioritized in the vaccine allocation mechanism by the government. Second, younger patients with better physical health felt less threatened by the pandemic and more inclined to wait for their most preferred vaccine, which was Pfizer in most cases. On the other hand, we see that patients without chronic illness, who received AstraZeneca have the highest average age. A potential explanation could be that elderly patient has a stronger time pressure and hence less likely to wait for alternative vaccines. Figure S3 shows a lot of variation among vaccine types and chronic illness status, which could be explained by prioritization of patients with certain jobs and the amount of time pressure patients experience to wait for an alternative vaccine.

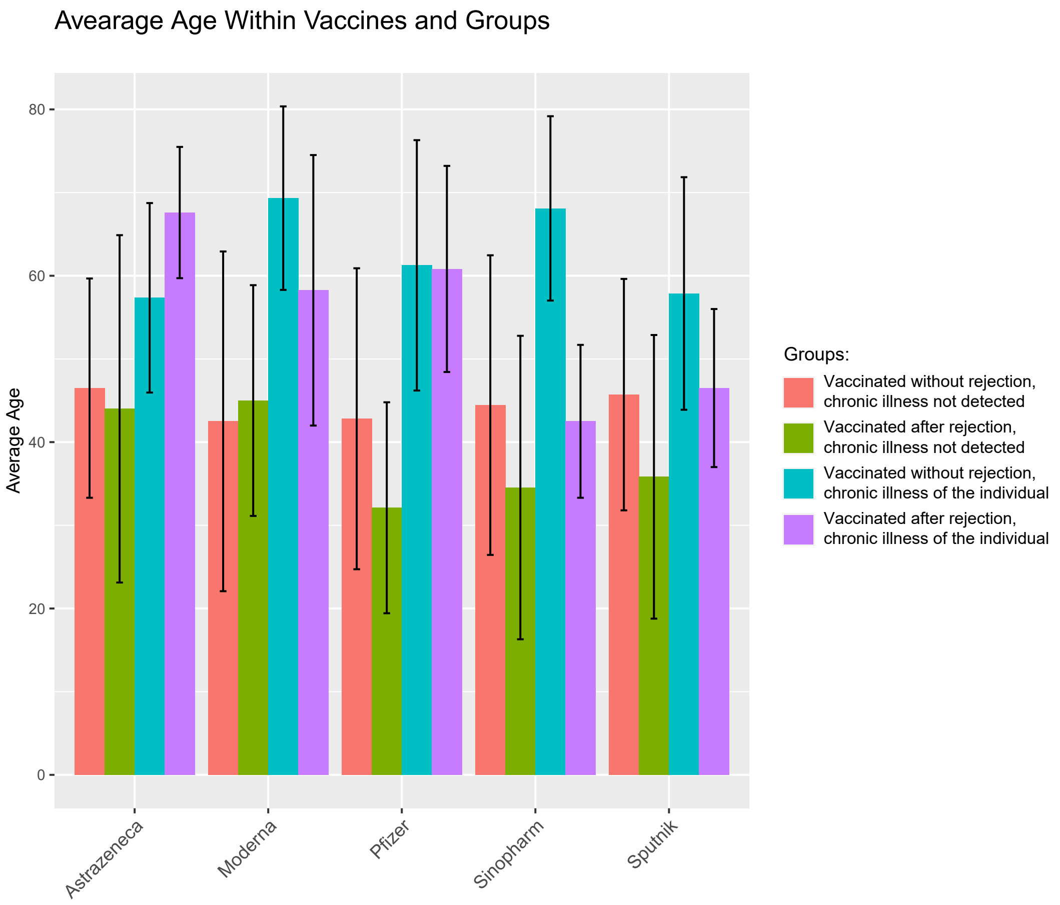

Figure S3: Average age distribution conditionally on vaccine type, chronic illness and rejection status. The black line shows 1 standard deviation.

Figure S4 shows the average week of the first vaccination among vaccine types, chronic illness and rejection status. We see that the average vaccination week tends to increase among those who rejected a vaccine regardless of chronic illness status and vaccination type. Let us point out the large variance in the vaccination date among patients who received Pfizer. This indicates that Pfizer was available for the longest time in Hungary. Also, the smaller values for AstraZeneca suggests that patients were more likely to receive AstraZeneca earlier, in February and early March. Figure S4 is in accordance with Figure S1 as they both show that vaccines became available for the most patient starting from the 14th week and patients with Pfizer have the largest variation in the time of vaccination.

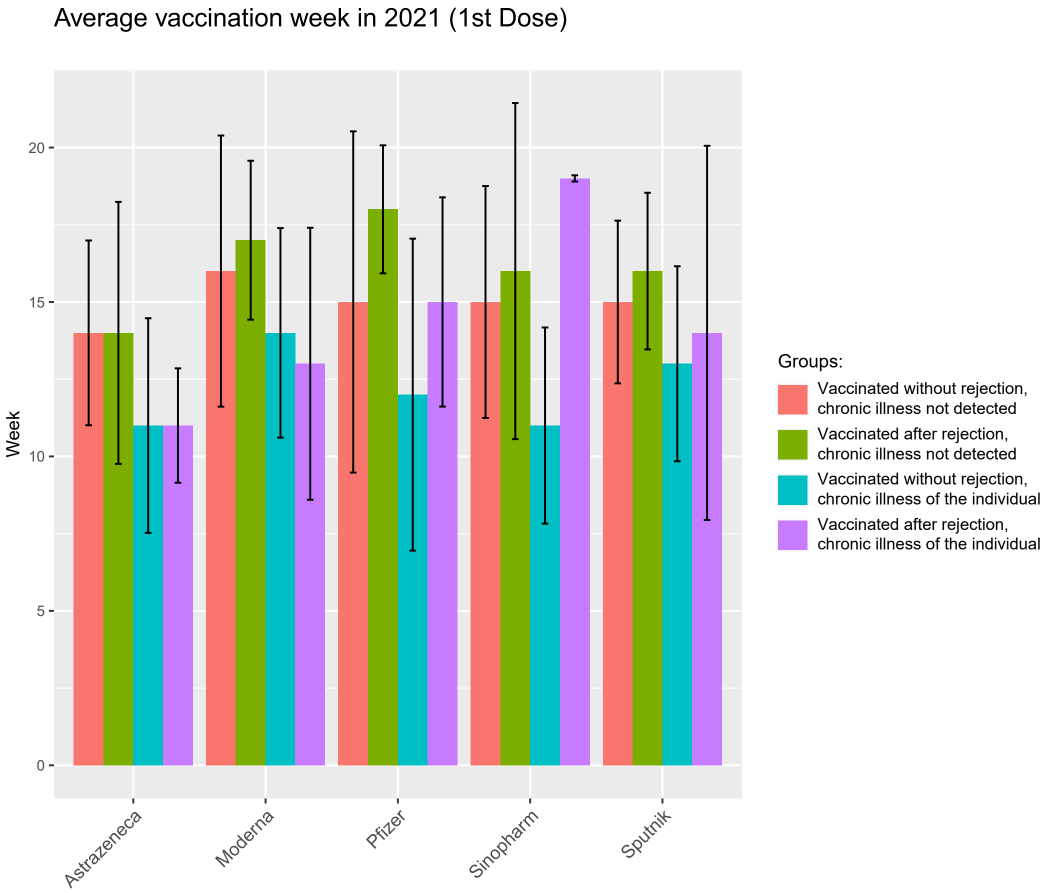

Figure S4: Average vaccination week distribution in 2021 conditionally on vaccine type, chronic illness and rejection status. The black line shows 1 standard deviation.

Supporting Information 2: Hesitancy by vaccine types

We investigate the hesitancy distributions by vaccine types. To do that, we apply the dependent variable of the multivariate regression analyses documented in Figure 3 and in Tables S4-S8. Table S1 illustrates that hesitancy is not significantly different across Pfizer and Moderna (lowest level of hesitancy). However, we find a significantly stronger hesitancy against Sputnik than against Pfizer. Hesitancy against AstraZeneca and Sinopharm are both significantly stronger than against Sputnik.

Chi2 tests suggest that the distributions of the above hesitancy measure by vaccine types are not independent from each other. Instead, these distributions are correlated as we show in Figure S5. We find strong correlation across Pfizer and Moderna hesitancy and also across Sputnik and Sinopharm hesitancy but not across these two groups of vaccines.

Table S1: Hesitancy by vaccine types

	Mean	95% Confidence Interval
Pfizer	0.040	[0.027; 0.052]
Moderna	0.046	[0.032; 0.059]
AstraZeneca	0.109	[0.089; 0.128]
Sputnik	0.068	[0.052; 0.083]
Sinopharm	0.111	[0.091; 0.130]

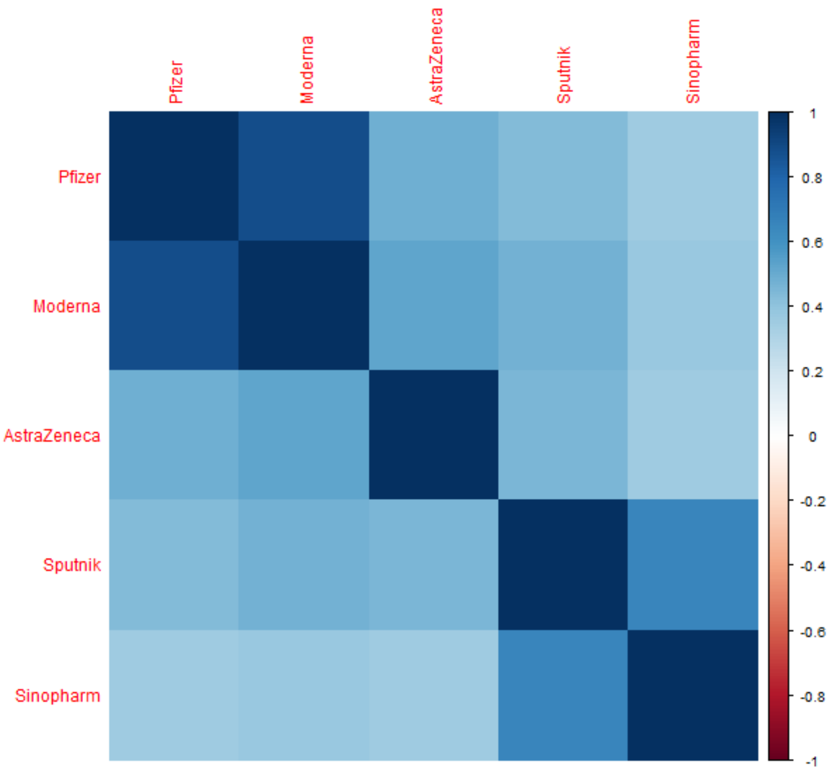

Figure S5: Correlation of hesitancy by vaccine types.

Supporting Information 3: Variable selection for the vaccine rejection analysis

Table S2: Lasso models on vaccine rejection

	Pfizer (1)	Moderna (2)	AstraZeneca (3)	Sputnik (4)	Sinopharm (5)	Variance (6)	Mean (7)	Min (8)	Max (9)	Any (10)
Age	x	x	x				x	x		x
Female		x	x	x						
City Cat. by Size	x	x	x	x						
University		x		x		x		x		
Smoking		x	x	x	x					x
Chronic Illness			x	x				x		
Acute Illness				x		x				
Worked Last Week				x		x		x		
Wealth Pre COVID-19		x								
Wealth Now		x	x	x	x	x	x	x		x
Adv. from Doct.	x	x	x	x		x	x		x	x
Adv. from Pol.				x		x				x
Adv. from Sci.	x	x	x	x		x		x	x	x
Adv. from Anti-vacc. Prop.	x	x	x	x		x		x		x
Adv. from Family								x		
Adv. from Friends				x						
Adv. from Celeb.						x				
Adv. from Journ.			x					x		
Online News			x	x	x	x		x		x

Note: Dependent variables: Pfizer and other vaccines: rating (1: "unacceptable"; 5: "best"); Variance/Mean/Min/Max: variance/mean/min/max of the ratings; UnacceptAny=1 if any vaccine rated as "unacceptable". Explanatory variables excluded from each lasso model: SourceSocialNetwork; SourcePress; SourceRadio; SourceTV; SourceFamily; SourceFriends; SourceOther (VaccineX: Takes advice regarding COVID-19 vaccination from X; SourceX: Source of information used to get to know about pandemic-related government measures)

Supporting Information 4: Variable description: summary statistics, correlation, VIF

Table S3: Summary statistics and VIF of variables in the linear probability model in Figure 3

Variable	Min	1st Qu.	Median	Mean	3rd Qu.	Max	VIF
Advice from Doctors	0.000	0.000	1.000	0.729	1.000	1.000	1.121
Advice from Scientists	0.000	0.000	1.000	0.595	1.000	1.000	1.168
Advice from Anti-vaccine Propagators	0.000	0.000	0.000	0.042	0.000	1.000	1.092
Advice from Politicians	0.000	0.000	0.000	0.065	0.000	1.000	1.236
Advice from Family	0.000	0.000	0.000	0.222	0.000	1.000	1.536
Advice from Friends	0.000	0.000	0.000	0.136	0.000	1.000	1.506
Advice from Journalists	0.000	0.000	0.000	0.017	0.000	1.000	1.586
Advice from Celebrities	0.000	0.000	0.000	0.015	0.000	1.000	1.583
Age	18.00	34.00	48.00	48.86	64.00	85.00	1.542
Female	0.00	0.00	1.00	0.53	1.00	1.00	1.075
University	0.000	.0.000	0.000	0.217	0.000	1.000	1.181
City Cat. by Size	1.000	1.000	2.000	2.296	3.000	4.000	1.073
Wealth Pre COVID-19	1.000	5.000	5.000	5.406	6.000	10.000	1.141
Smoking	1.000	1.000	2.000	2.208	4.000	4.000	1.114
Chronic Illness	0.000	0.000	0.000	0.406	1.000	1.000	1.403
Covid-19 Previously	0.000	0.000	0.000	0.095	0.000	1.000	1.157
Serious Covid-19 Previously	0.00	0.00	0.00	0.01	0.00	.00	1.123

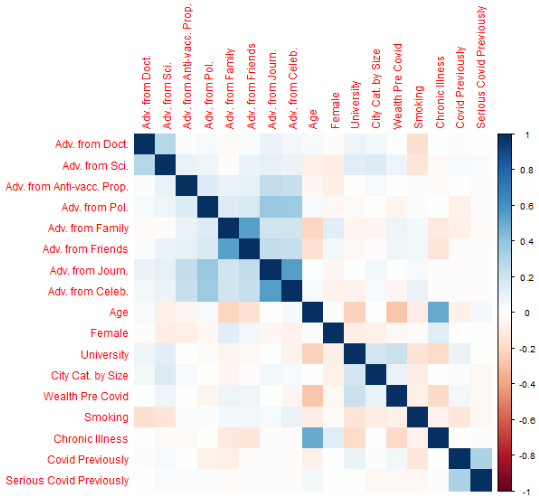

Figure S6: Pearson correlation coefficients of variables in the linear probability model in Figure 3

Supporting Information 5: Alternative specifications of the vaccine rejection analysis

Table S4: Linear probability models on evaluating a vaccine unacceptable. Results depicted in Figure 3

	Dependent variable: Evaluating a vaccine unacceptable				
	Pfizer	Moderna	AstraZeneca	Sputnik	Sinopharm
	(1)	(2)	(3)	(4)	(5)
Advice from Doctors	−0.057***	−0.061***	−0.029	−0.026	−0.054**
	(0.018)	(0.019)	(0.023)	(0.019)	(0.025)
Advice from Scientists	−0.035**	−0.026*	−0.054**	−0.017	0.008
	(0.014)	(0.014)	(0.022)	(0.017)	(0.021)
Advice from Anti-Vaccine Propagators	0.202***	0.182***	0.117*	0.062	0.054
	(0.064)	(0.066)	(0.066)	(0.056)	(0.064)
Advice from Politicians	−0.029	−0.035	−0.064**	−0.076***	−0.084***
	(0.023)	(0.023)	(0.031)	(0.014)	(0.030)
Advice from Family	−0.043***	−0.038**	0.028	−0.019	−0.058**
	(0.017)	(0.019)	(0.032)	(0.026)	(0.028)
Advice from Friends	−0.007	−0.007	−0.015	−0.025	−0.012
	(0.020)	(0.024)	(0.039)	(0.030)	(0.034)
Advice from Journalists	−0.071	−0.032	0.023	−0.008	0.089
	(0.049)	(0.060)	(0.100)	(0.058)	(0.138)
Advice from Celebrities	0.073	0.116	0.047	0.129	0.114
	(0.092)	(0.102)	(0.122)	(0.103)	(0.148)
Age	−0.001**	−0.001**	−0.003***	−0.0003	−0.0001
	(0.0004)	(0.0005)	(0.001)	(0.001)	(0.001)
Female	0.006	0.001	0.045**	−0.005	0.005
	(0.013)	(0.014)	(0.020)	(0.017)	(0.021)
University	−0.021	−0.026*	−0.054**	−0.026	0.002
	(0.015)	(0.015)	(0.024)	(0.018)	(0.024)
City Category by Size	0.0002	−0.005	−0.005	−0.004	0.010
	(0.005)	(0.006)	(0.009)	(0.008)	(0.010)
Smoking	0.004	0.005	0.004	0.011	0.020**
	(0.005)	(0.006)	(0.008)	(0.007)	(0.008)
Chronic Illness	−0.004	−0.004	0.008	−0.011	0.002
	(0.016)	(0.015)	(0.023)	(0.021)	(0.025)
COVID-19 Previously	0.007	0.002	−0.010	−0.034	−0.045
	(0.023)	(0.023)	(0.035)	(0.022)	(0.031)
Serious COVID-19 Previously	0.151	0.149	0.031	−0.031	0.037
	(0.109)	(0.109)	(0.094)	(0.028)	(0.101)
Constant	0.146***	0.166***	0.314***	0.120**	0.095*
	(0.038)	(0.041)	(0.054)	(0.047)	(0.055)
Observations	999	999	999	999	999
R^2	0.095	0.080	0.070	0.026	0.028
Adjusted R^2	0.080	0.065	0.055	0.010	0.012
Note:					*p<0.1; **p<0.05; ***p<0.01

Table S5: Linear probability models on evaluating a vaccine unacceptable with standardized variables

	Dependent variable: Evaluating a vaccine unacceptable				
	Pfizer	Moderna	AstraZeneca	Sputnik	Sinopharm
	(1)	(2)	(3)	(4)	(5)
Advice from Doctors	−0.130***	−0.130***	−0.041	−0.046	−0.076**
	(0.040)	(0.039)	(0.033)	(0.034)	(0.035)
Advice from Scientists	−0.087**	−0.062*	−0.085**	−0.034	0.013
	(0.034)	(0.034)	(0.034)	(0.034)	(0.033)
Advice from Anti-Vaccine Propagators	0.207***	0.174***	0.076*	0.049	0.035
	(0.066)	(0.063)	(0.043)	(0.045)	(0.041)
Advice from Politicians	−0.036	−0.041	−0.051**	−0.074***	−0.066***
	(0.029)	(0.027)	(0.024)	(0.014)	(0.023)
Advice from Family	−0.092***	−0.075**	0.038	−0.031	−0.077**
	(0.035)	(0.038)	(0.043)	(0.043)	(0.037)
Advice from Friends	−0.011	−0.011	−0.016	−0.034	−0.013
	(0.035)	(0.039)	(0.043)	(0.041)	(0.037)
Advice from Journalists	−0.047	−0.020	0.010	−0.004	0.037
	(0.032)	(0.037)	(0.041)	(0.030)	(0.057)
Advice from Celebrities	0.045	0.067	0.018	0.062	0.044
	(0.057)	(0.059)	(0.047)	(0.050)	(0.057)
Age	−0.089**	−0.086**	−0.200***	−0.019	−0.003
	(0.041)	(0.041)	(0.039)	(0.042)	(0.040)
Female	0.016	0.002	0.072**	−0.010	0.008
	(0.033)	(0.033)	(0.032)	(0.034)	(0.033)
University	−0.044	−0.051*	−0.072**	−0.042	0.003
	(0.031)	(0.029)	(0.032)	(0.029)	(0.032)
City Category by Size	0.001	−0.027	−0.018	−0.017	0.033
	(0.028)	(0.029)	(0.029)	(0.033)	(0.034)
Smoking	0.023	0.028	0.018	0.054	0.081**
	(0.035)	(0.035)	(0.034)	(0.035)	(0.034)
Chronic Illness	−0.009	−0.009	0.013	−0.022	0.004
	(0.039)	(0.036)	(0.036)	(0.040)	(0.040)
COVID-19 Previously	0.010	0.003	−0.009	−0.040	−0.042
	(0.034)	(0.032)	(0.033)	(0.026)	(0.029)
Serious COVID-19 Previously	0.076	0.071	0.010	−0.012	0.012
	(0.055)	(0.052)	(0.030)	(0.011)	(0.032)
Constant	0.0003	0.0003	0.0001	0.0001	0.0003
	(0.030)	(0.031)	(0.031)	(0.031)	(0.031)
Observations	999	999	999	999	999
R^2	0.095	0.080	0.070	0.026	0.028
Adjusted R^2	0.080	0.065	0.055	0.010	0.012

Note: *p<0.1; **p<0.05; ***p<0.01

Table S6: Logistic regression on evaluating a vaccine unacceptable

	Dependent variable: Evaluating a vaccine unacceptable				
	Pfizer	Moderna	AstraZeneca	Sputnik	Sinopharm
	(1)	(2)	(3)	(4)	(5)
Advice from Doctors	−1.262***	−1.211***	−0.298	−0.381	−0.510**
	(0.396)	(0.358)	(0.229)	(0.266)	(0.217)
Advice from Scientists	−1.085**	−0.682*	−0.573**	−0.295	0.039
	(0.439)	(0.379)	(0.236)	(0.271)	(0.219)
Advice from Anti-Vaccine Propagators	2.838***	2.349***	0.974**	0.938	0.495
	(0.560)	(0.549)	(0.459)	(0.588)	(0.517)
Advice from Politicians	−1.122	−1.228	−0.939	−3.267***	−1.474*
	(1.051)	(1.016)	(0.635)	(0.844)	(0.834)
Advice from Family	−1.441**	−1.041*	0.138	−0.429	−0.722**
	(0.629)	(0.554)	(0.278)	(0.441)	(0.363)
Advice from Friends	−0.479	−0.343	−0.066	−0.461	−0.176
	(0.718)	(0.672)	(0.338)	(0.609)	(0.444)
Advice from Journalists	−15.071***	−0.544	0.380	0.790	1.221
	(1.224)	(1.644)	(1.211)	(0.901)	(1.515)
Advice from Celebrities	3.350***	2.947***	0.910	2.621**	1.347
	(0.886)	(0.905)	(1.341)	(1.018)	(1.390)
Age	−0.029**	−0.026**	−0.037***	−0.004	−0.001
	(0.014)	(0.013)	(0.008)	(0.009)	(0.007)
Female	0.122	−0.006	0.467**	−0.060	0.046
	(0.395)	(0.355)	(0.231)	(0.269)	(0.215)
University	−0.857	−0.914*	−0.525*	−0.513	0.033
	(0.555)	(0.518)	(0.300)	(0.392)	(0.265)
City Category by Size	−0.021	−0.179	−0.086	−0.070	0.105
	(0.165)	(0.163)	(0.104)	(0.134)	(0.104)
Smoking	0.133	0.127	0.042	0.176*	0.204**
	(0.140)	(0.130)	(0.088)	(0.104)	(0.083)
Chronic Illness	−0.080	−0.092	0.103	−0.203	0.017
	(0.481)	(0.409)	(0.276)	(0.338)	(0.262)
COVID-19 Previously	0.097	0.005	−0.090	−0.811	−0.558
	(0.610)	(0.592)	(0.394)	(0.667)	(0.459)
Serious COVID-19 Previously	1.941**	1.727*	0.321	−13.129***	0.395
	(0.888)	(0.918)	(1.024)	(0.828)	(1.235)
Constant	−0.840	−0.557	−0.097	−1.892**	−2.295***
	(0.933)	(0.850)	(0.550)	(0.741)	(0.582)
Observations	999	999	999	999	999
Akaike Inf. Crit.	292.334	340.300	654.138	499.692	701.087

Note: *p<0.1; **p<0.05; ***p<0.01

Table S7: Linear probability models on evaluating a vaccine unacceptable with individual subjective wealth

	Dependent variable: Evaluating a vaccine unacceptable				
	Pfizer	Moderna	AstraZeneca	Sputnik	Sinopharm
	(1)	(2)	(3)	(4)	(5)
Advice from Doctors	−0.058***	−0.062***	−0.029	−0.027	−0.054**
	(0.018)	(0.019)	(0.023)	(0.019)	(0.025)
Advice from Scientists	−0.034**	−0.026*	−0.054**	−0.017	0.009
	(0.014)	(0.014)	(0.021)	(0.017)	(0.021)
Advice from Anti-Vaccine Propagators	0.202***	0.182***	0.117*	0.061	0.054
	(0.064)	(0.066)	(0.066)	(0.056)	(0.064)
Advice from Politicians	−0.030	−0.037*	−0.065**	−0.079***	−0.088***
	(0.022)	(0.022)	(0.030)	(0.015)	(0.030)
Advice from Family	−0.043***	−0.037**	0.029	−0.018	−0.057**
	(0.017)	(0.019)	(0.032)	(0.026)	(0.028)
Advice from Friends	−0.006	−0.006	−0.014	−0.024	−0.011
	(0.020)	(0.024)	(0.039)	(0.030)	(0.034)
Advice from Journalists	−0.070	−0.030	0.024	−0.006	0.092
	(0.049)	(0.060)	(0.100)	(0.058)	(0.138)
Advice from Celebrities	0.071	0.114	0.046	0.124	0.109
	(0.091)	(0.102)	(0.122)	(0.100)	(0.145)
Age	−0.001**	−0.001**	−0.004***	−0.0004	−0.0003
	(0.0005)	(0.0005)	(0.001)	(0.001)	(0.001)
Female	0.006	0.0004	0.045**	−0.005	0.004
	(0.013)	(0.014)	(0.020)	(0.017)	(0.021)
University	−0.019	−0.023	−0.053**	−0.021	0.008
	(0.014)	(0.015)	(0.024)	(0.018)	(0.025)
City Category by Size	0.001	−0.005	−0.005	−0.003	0.011
	(0.005)	(0.006)	(0.009)	(0.008)	(0.010)
Wealth pre COVID-19	−0.004	−0.005	−0.003	−0.009*	−0.011
	(0.004)	(0.004)	(0.006)	(0.005)	(0.007)
Smoking	0.003	0.004	0.004	0.010	0.019**
	(0.005)	(0.006)	(0.008)	(0.007)	(0.008)
Chronic Illness	−0.005	−0.005	0.008	−0.013	−0.0002
	(0.016)	(0.015)	(0.023)	(0.021)	(0.025)
COVID-19 Previously	0.008	0.003	−0.009	−0.032	−0.043
	(0.023)	(0.023)	(0.035)	(0.022)	(0.031)
Serious COVID-19 Previously	0.148	0.146	0.029	−0.036	0.030
	(0.110)	(0.110)	(0.094)	(0.027)	(0.100)
Constant	0.175***	0.199***	0.332***	0.178***	0.162**
	(0.051)	(0.053)	(0.068)	(0.060)	(0.073)
Observations	999	999	999	999	999
R^2	0.096	0.082	0.070	0.029	0.031
Adjusted R^2	0.081	0.066	0.054	0.013	0.014

Note: *p<0.1; **p<0.05; ***p<0.01

Table S8: Linear probility models on evaluating a vaccine unacceptable among those individuals who had stable vaccine assessment over the period

	Dependent variable: Evaluating a vaccine unacceptable				
	Pfizer	Moderna	AstraZeneca	Sputnik	Sinopharm
	(1)	(2)	(3)	(4)	(5)
Advice from Doctors	−0.052***	−0.052***	−0.018	−0.022	−0.038
	(0.018)	(0.018)	(0.023)	(0.020)	(0.024)
Advice from Scientists	−0.034**	−0.025*	−0.042*	−0.011	0.006
	(0.014)	(0.014)	(0.022)	(0.018)	(0.022)
Advice from Anti-Vaccine Propagators	0.175***	0.143**	0.121*	0.040	0.012
	(0.065)	(0.064)	(0.073)	(0.055)	(0.061)
Advice from Politicians	−0.026	−0.031	−0.060**	−0.076***	−0.081**
	(0.024)	(0.022)	(0.027)	(0.014)	(0.031)
Advice from Family	−0.041**	−0.033*	0.029	−0.014	−0.055**
	(0.017)	(0.019)	(0.031)	(0.027)	(0.028)
Advice from Friends	−0.006	−0.005	−0.051	−0.023	−0.027
	(0.021)	(0.024)	(0.035)	(0.032)	(0.032)
Advice from Journalists	−0.068	−0.027	0.090	−0.008	0.119
	(0.050)	(0.059)	(0.089)	(0.058)	(0.162)
Advice from Celebrities	0.082	0.120	−0.102	0.131	0.122
	(0.104)	(0.103)	(0.064)	(0.102)	(0.158)
Age	−0.001**	−0.001*	−0.003***	−0.0002	−0.0001
	(0.0005)	(0.0005)	(0.001)	(0.001)	(0.001)
Female	0.009	0.002	0.048**	−0.006	0.013
	(0.013)	(0.014)	(0.020)	(0.017)	(0.021)
University	−0.019	−0.022	−0.045*	−0.017	0.004
	(0.015)	(0.015)	(0.025)	(0.019)	(0.025)
City Category by Size	0.001	−0.005	−0.001	−0.005	0.008
	(0.005)	(0.006)	(0.009)	(0.008)	(0.010)
Smoking	0.004	0.007	0.011	0.012*	0.019**
	(0.005)	(0.006)	(0.008)	(0.007)	(0.008)
Chronic Illness	−0.004	−0.004	−0.0003	−0.005	−0.003
	(0.016)	(0.015)	(0.023)	(0.021)	(0.026)
COVID-19 Previously	0.012	0.006	−0.029	−0.032	−0.040
	(0.024)	(0.023)	(0.033)	(0.024)	(0.033)
Serious COVID-19 Previously	0.175	0.151	0.079	−0.031	0.039
	(0.120)	(0.111)	(0.101)	(0.028)	(0.101)
Constant	0.136***	0.143***	0.263***	0.103**	0.093*
	(0.037)	(0.039)	(0.052)	(0.046)	(0.055)
Observations	962	983	898	951	946
R^2	0.082	0.064	0.073	0.022	0.023
Adjusted R^2	0.067	0.048	0.056	0.005	0.007

Note: *p<0.1; **p<0.05; ***p<0.01

Supporting Information 6: Summary statistics for vaccine assessment matrices

Table S9: Summary statistics for Figure 4**A**: Accepted first vaccine

Accepted Vaccine	Rated Vaccine	Observations	Mean	Standard Deviation
AstraZeneca	Pfizer	121	4.17	1.03
AstraZeneca	Moderna	121	3.94	1.00
AstraZeneca	AstraZeneca	121	3.84	1.00
AstraZeneca	Sputnik	121	3.56	1.12
AstraZeneca	Sinopharm	121	3.30	1.24
Moderna	Pfizer	35	4.32	1.06
Moderna	Moderna	35	4.28	0.92
Moderna	AstraZeneca	35	3.19	1.04
Moderna	Sputnik	35	3.59	1.18
Moderna	Sinopharm	35	3.18	1.36
Pfizer	Pfizer	202	4.58	0.70
Pfizer	Moderna	202	4.05	1.00
Pfizer	AstraZeneca	202	3.15	1.17
Pfizer	Sputnik	202	3.46	1.19
Pfizer	Sinopharm	202	3.07	1.27
Sinopharm	Pfizer	146	3.99	1.17
Sinopharm	Moderna	146	3.82	1.13
Sinopharm	AstraZeneca	146	3.36	1.23
Sinopharm	Sputnik	146	3.75	1.10
Sinopharm	Sinopharm	146	3.90	1.15
Sputnik	Pfizer	123	4.13	1.08
Sputnik	Moderna	123	3.94	1.07
Sputnik	AstraZeneca	123	3.14	1.09
Sputnik	Sputnik	123	4.02	1.05
Sputnik	Sinopharm	123	3.44	1.17

Table S10: Summary statistics for Figure 4**B**: Rejected at least 1 vaccine; Grouped by accepted vaccine

Accepted Vaccine	Rated Vaccine	Observations	Mean	Standard Deviation
AstraZeneca	Pfizer	11	4.30	1.25
AstraZeneca	Moderna	11	3.78	1.20
AstraZeneca	AstraZeneca	11	3.91	0.83
AstraZeneca	Sputnik	11	2.88	0.83
AstraZeneca	Sinopharm	11	2.12	0.83
Moderna	Pfizer	10	4.88	0.35
Moderna	Moderna	10	5.00	0.00
Moderna	AstraZeneca	10	3.12	0.99
Moderna	Sputnik	10	3.75	1.04
Moderna	Sinopharm	10	2.12	1.36
Pfizer	Pfizer	58	4.77	0.54
Pfizer	Moderna	58	4.36	0.86
Pfizer	AstraZeneca	58	2.70	1.35
Pfizer	Sputnik	58	2.92	1.25
Pfizer	Sinopharm	58	2.73	1.39
Sinopharm	Pfizer	16	3.78	1.20
Sinopharm	Moderna	16	3.00	1.00
Sinopharm	AstraZeneca	16	2.00	0.87
Sinopharm	Sputnik	16	3.22	0.83
Sinopharm	Sinopharm	16	4.18	0.87
Sputnik	Pfizer	13	3.90	1.10
Sputnik	Moderna	13	3.75	1.16
Sputnik	AstraZeneca	13	2.50	1.27
Sputnik	Sputnik	13	4.08	0.79
Sputnik	Sinopharm	13	3.50	1.18

Table S11: Summary statistics for Figure 4**C**: Rejected at least 1 vaccine; Grouped by assigned vaccine

Assigned Vaccine	Rated Vaccine	Observations	Mean	Standard Deviation
AstraZeneca	Pfizer	28	4.26	1.14
AstraZeneca	Moderna	28	3.95	1.12
AstraZeneca	AstraZeneca	28	2.20	1.08
AstraZeneca	Sputnik	28	3.42	1.10
AstraZeneca	Sinopharm	28	3.24	1.51
Moderna	Pfizer	2	4.50	0.71
Moderna	Moderna	2	2.00	0.00
Moderna	AstraZeneca	2	2.50	0.71
Moderna	Sputnik	2	2.50	0.71
Moderna	Sinopharm	2	4.00	0.00
Pfizer	Pfizer	6	3.25	1.71
Pfizer	Moderna	6	3.25	1.71
Pfizer	AstraZeneca	6	2.50	1.00
Pfizer	Sputnik	6	4.00	0.82
Pfizer	Sinopharm	6	4.00	1.22
Sinopharm	Pfizer	44	4.76	0.43
Sinopharm	Moderna	44	4.49	0.70
Sinopharm	AstraZeneca	44	3.24	1.35
Sinopharm	Sputnik	44	3.28	1.14
Sinopharm	Sinopharm	44	2.62	1.10
Sputnik	Pfizer	26	4.61	0.72
Sputnik	Moderna	26	4.06	1.00
Sputnik	AstraZeneca	26	2.78	1.31
Sputnik	Sputnik	26	2.47	1.12
Sputnik	Sinopharm	26	2.50	1.51

Supporting Information 7: Dynamics of vaccine assessment

In the nationally representative survey, individuals are asked whether they have changed their opinion about each of the available vaccine types over the past few months. In Table S12, we show the number of observations who declared a negative and positive change, respectively.

Table S12: Number of respondents who changed their attitudes towards vaccines

	Negative change	Positive change
Pfizer	19	18
Moderna	5	11
AstraZeneca	90	10
Sputnik	17	31
Sinopharm	34	19

In Table S13, we consider those individuals who accepted a vaccine that they did not rate the highest among the available vaccines. Based on the accepted vaccine type, we calculated the ratio of individuals who rate an other vaccine the highest. For instance, among the 9 observations who accepted Pfizer and did not rate Pfizer as their most preferred vaccine there were 2 individuals, who rate Moderna as the most preferred vaccine, and hence we obtained 0.22. As individuals were allowed to give the highest rating to multiple vaccines, we see that the sum of the "Best" ratings must be weakly greater than 1. Let us point out that AstraZeneca group has the most observations, who rate another vaccine the highest, and then Sputnik and Sinopharm follows. We can also see a strong preference toward Pfizer among those who did not accept their most preferred vaccine, which suggests the limited supply of Pfizer in the observed period in Hungary.

Table S13: Distribution of the most preferred vaccine types when the accepted vaccine is not the most preferred.

Accepted vaccine	Observations	Pfizer	Moderna	AstraZeneca	Sputnik	Sinopharm
Pfizer	9	-	0.22	0.11	0.78	0.44
Moderna	3	1.00	-	0.00	0.33	0.00
AstraZeneca	40	0.88	0.25	-	0.10	0.10
Sputnik	29	0.93	0.48	0.07	-	0.10
Sinopharm	29	0.79	0.41	0.17	0.17	-

The representative survey asks individuals to estimate the number of weeks that they would be willing to wait to get their most preferred vaccine. In Table S14, we grouped individuals based on whether they rejected any vaccines and they rate the accepted vaccine the highest. We see that those who accepted a vaccine that they do not rate the highest estimated the expected number of weeks until the best vaccine to be 2.34 and 2.57 weeks on average if not rejected any and rejected at least 1 vaccine, respectively. We see that these estimations are relatively close to 2.76, which is the mean of the actual number weeks that the those individuals waited, who rejected a vaccine and ended up accepting a vaccine that they rate the highest.

Table S14: Expected and actual waiting time distribution based on rejecting any vaccines and rating the accepted vaccine the highest.

Rejected any	Highest rating	Observations	Mean expected (weeks)	Mean actual (weeks)	90% CI expected (weeks)	90% CI actual (weeks)
0	0	101	2.34	-	(1.90,2.78)	(-,-)
0	1	526	0.29	-	(0.21,0.36)	(-,-)
1	0	9	2.57	0.83	(0.58,4.56)	(0.11,1.56)
1	1	99	0.25	2.76	(0.11,0.39)	(2.20,3.32)

In Table S15, we grouped individuals, who accepted a vaccine that they do not rate the highest and individuals who were first offered and rejected that vaccine, and ended up getting a vaccine that they rate the highest. First, notice that the waiting times are not homogeneous among the vaccine types. For instance, the mean expected and the mean actual waiting times for Sinopharm are 3.46 and 3.78 weeks,

respectively, while for Pfizer we have only 1.20 and 1.00. Let us also highlight the trend that AstraZeneca has a higher acceptance to rejection ($\frac{40}{25}$) ratio than the one that Sputnik or Sinopharm has ($\frac{29}{23}$ and $\frac{29}{41}$, respectively). This could be explained by that the AstraZeneca group estimated the expected waiting time to be longer than it actually was, while we see the opposite for Sputnik and Sinopharm.

Table S15: Expected and actual waiting times for accepting and rejecting a vaccine that is not the most preferred.

Vaccine type	Observations Accept	Observations Reject	Mean expected Accept (week)	Mean actual Reject (week)	90% CI expected Accept (week)	90% CI actual Reject (week)
AstraZeneca	40	25	2.61	1.94	(1.87,3.36)	(1.47,2.42)
Moderna	3	1	0.00	2.00	(-,-)	(-,-)
Pfizer	9	5	1.20	1.00	(0.09,2.31)	(-0.35,2.35)
Sinopharm	29	41	3.46	3.78	(2.37,4.55)	(2.60,4.96)
Sputnik	29	23	1.61	2.58	(1.04,2.19)	(1.37,3.80)

Supporting Information 8: Co-acceptance of vaccines among non-vaccinated

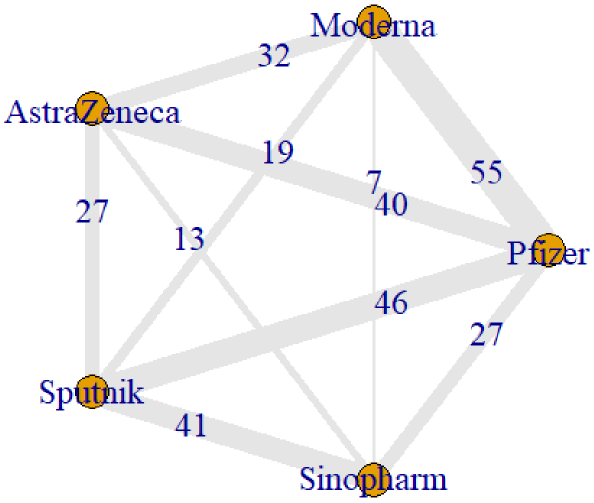

Figure S7: Co-acceptance of vaccines. Edge labels reflect to the number of individuals who accept both vaccines included. We only include those individuals to create this network who accept 2 or 3 vaccines.
